# Experimental Verification of the Pumping Effect Caused by the Micro-Tapered Hole in a Piezoelectric Atomizer

**DOI:** 10.3390/s18072311

**Published:** 2018-07-17

**Authors:** Jianhui Zhang, Qiufeng Yan, Jun Huang, Chuanyu Wu

**Affiliations:** 1 School of Mechanical and Electric Engineering, Guangzhou University, Guangzhou 510006, China; 2 State Key Laboratory of Mechanics and Control of Mechanical Structures, Nanjing University of Aeronautics and Astronautics, Nanjing 211106, China; yanqf@nuaa.edu.cn; 3 Research Center of Fluid Machinery Engineering and Technology, Jiangsu University, Zhenjiang 212013, China; huangjun@ujs.edu.cn; 4 College of Mechanical Engineering and Automation, Zhejiang Sci-Tech University, Hangzhou 310018, China; cywu@zstu.edu.cn

**Keywords:** micro-tapered aperture, pumping effect, atomization rate

## Abstract

In this study, we examined the use of a dynamic micro-tapered hole as a micro-scale tapered flow tube valveless piezoelectric pump. Firstly, we obtained photographs of a micro-tapered hole by using an environmental scanning electron microscope (ESEM). Then, we explained the pump effect of the micro-tapered hole, and derived the atomization rate equation. Furthermore, we reported an atomization rate measurement experiment that eliminated the atomization caused by a pressure increase, and demonstrated that a change in the volume of a micro-tapered hole could produce atomization. The experimental results indicate that, under the same voltage, the forward atomization rate is much higher than the reverse atomization rate and that the atomization rate increases with the micro-tapered hole volume. The experimental results show that the atomization of the micro-tapered aperture atomizer is caused by its pumping effect. Moreover, the flow resistance and volume of the micro-tapered hole can affect the atomization rate.

## 1. Introduction

Ultrafine atomization is an important technique in inhalation therapy [1,2], spray drying [3], mass spectrometry [4], printed circuits [5], three-dimensional (3D) prototyping [6], and precise surface coating [7,8].

The micro-tapered aperture atomizer is a new device used for ultrafine atomization. This type of atomizer uses a micro-tapered hole on the metal plate of a piezoelectric vibrator; and its structure does not require a variable-volume liquid chamber during atomization, thereby simplifying the structure and providing better control of the atomization process. The micro-tapered aperture atomizer has many advantages, including its simple structure, low cost, portability and straightforward integration, and uniform particle size distribution, which have attracted the attention of several researchers [9,10,11,12]. However, research in this area has mainly focused on the structure and performance of the micro-tapered aperture atomizer, whereas few studies have considered the atomization mechanisms. Lu [13,14] reviewed the operation of this type of atomizer, and the basic process is described as follows: The liquid is pinched off as droplets pass through the cone nozzle during forward vibration, and then adheres to the orifice by capillary force during backward vibration. Driven by a high-frequency piezoelectric ceramic ring, the liquid is repeatedly pinched off and attached, resulting in atomization.

According to the working mechanism of the atomizer proposed by Lu, the atomization rate in the forward direction should be consistent with that in the reverse direction under the same voltage. In a previous study by our group [15,16], it was found that under the same voltage, the forward atomization rate is much higher than the reverse atomization rate. Therefore, the concept of a dynamic cone angle, proposed in the previous study by our group, has been demonstrated. The volume variation in the liquid chamber and the micro-tapered hole, and the difference between the forward direction flow resistance and the reverse direction flow resistance of the micro-tapered hole are considered as the causes of atomization in the atomizer. Moreover, experimental verification has been carried out.

In further research, it was found that the actual measured atomization rate was caused by the volume variation in the micro-tapered hole and piezoelectric vibrator. In addition, we were unable to directly measure the atomization caused by the volume variation in the micro-tapered hole. The atomization rate cannot directly reflect the role of the pumping effect caused by the volume variation in the micro-tapered hole in the atomization process.

In this research, we designed a new experiment to directly measure the atomization rate caused by the volume variation in the micro-tapered hole. Photographs of the micro-tapered hole were recorded using an environmental scanning electron microscope (ESEM). The volume variation in the micro-tapered hole was regarded as the volume variation in the piezoelectric vibrator of the valveless piezoelectric pump, and the micro-tapered hole approximates the conical flow path of the valveless piezoelectric pump. The flow resistance of the micro-tapered hole was analyzed, and the average forward and reverse flow resistances and the atomization rate equation were deduced. The forward and reverse atomization rates were measured, and the results suggested that the micro-tapered hole and its variation played an important role in the atomization process. Measuring the atomization rate of three single-diameter atomizers of different sizes, led to the finding that the volume of the micro-tapered hole also plays an important role in atomization.

## 2. Theoretical Analysis

### 2.1. The Structure of the Micro-Tapered Aperture

**Figure 1:**
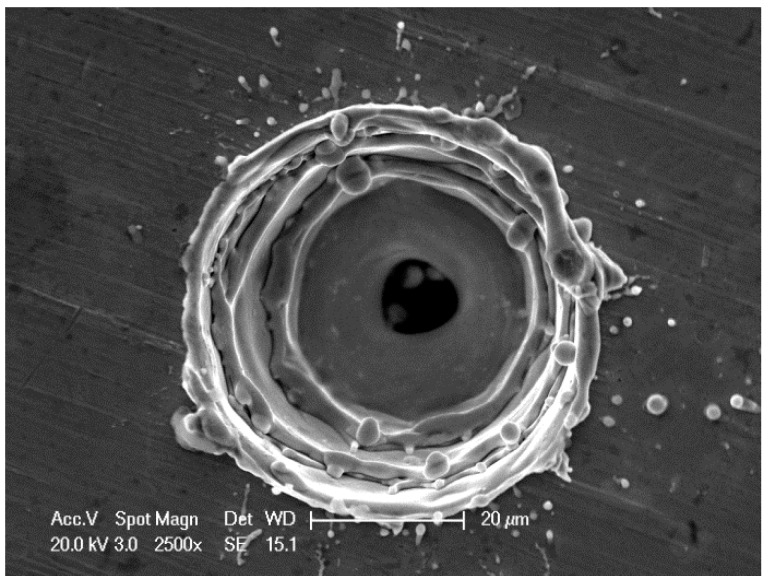
Environmental scanning electron microscope (ESEM) photograph of the micro-tapered hole.

Figure 1 presents a photograph of a micro-tapered hole using an ESEM, which clearly reveals the hole’s structure. The micro-tapered hole can be observed as a series of three micro-tapered holes.

### 2.2. The Pumping Effect of the Micro-Tapered Aperture

**Figure 2:**
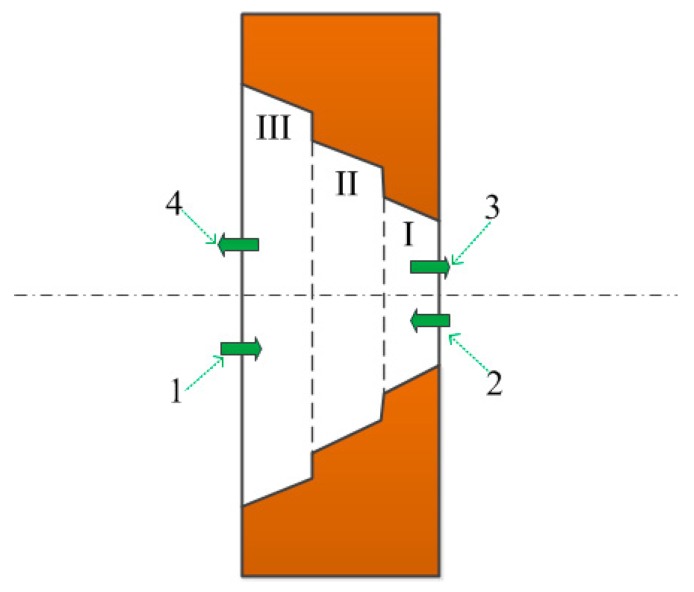
Flow channel of the micro-tapered hole.

Figure 2 shows the flow channel of the micro-tapered hole. According to the photographs obtained by ESEM, the flow channel of the micro-tapered hole is divided into Sections I, II, and III, from right to left. Here, we define the flow of liquid from III to I as the forward direction of the liquid flow, and the flow of liquid from I to III as the reverse direction of theliquid flow.

During operation, the volume of the micro-tapered hole will vary periodically due to the alternating current (AC) voltage. When the volume of the micro-tapered hole increases, the pressure in the hole decreases. The liquid flows from the liquid chamber (along arrow 1) and the external environment (along arrow 2) into the interior of the micro-tapered hole. When the volume of the micro-tapered hole decreases, the pressure in the hole increases. The liquid flows from the inside of the micro-tapered hole to the external environment (along arrow 3) and the liquid chamber (along arrow 4). Due to the difference between the forward and reverse flow resistances, the liquid flow rates into and out of the micro-tapered hole from the two directions are different. Under AC voltage, a macroscopic one-way flow is formed.

Olsson [17] compared the results obtained from numerical calculations for conical flows and those mentioned by White [18], and found that the flow resistances in the nozzle/diffuser elements at the micro-scale are similar to those at the macro-scale. The micro-tapered aperture, as shown in Figure 2, consists of parts I, II, and III, and the micro-tapered aperture is machined on the substrate of the piezoelectric vibrator. Under AC voltage, the volume of the micro-tapered aperture changes continuously, and because of the difference of the forward and reverse flow resistances, a micro-pump can be formed to make the liquid flow in one way. 

**Figure 3:**
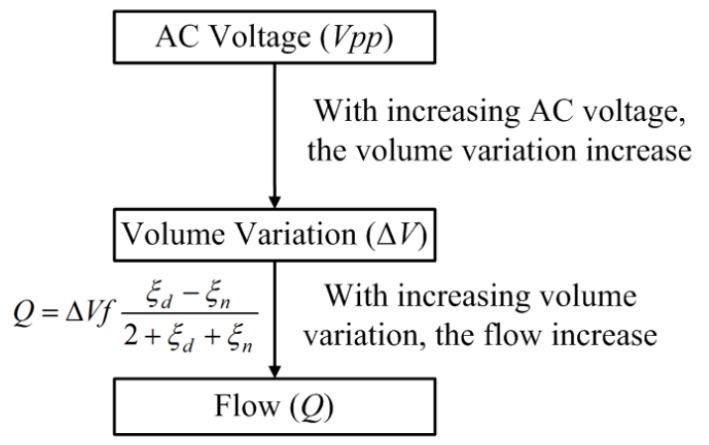
Conceptual plot of alternating current (AC) voltage, volume variation, and flow.

Figure 3 shows the conceptual plot of the AC voltage, volume variation, and flow. The AC voltage affects the volume variation of the micro-tapered aperture, which in turn affects the flow. Therefore, in this study, a dynamic micro-tapered hole can be considered a micro-scale tapered flow tube valveless piezoelectric pump. The volume variation in the micro-tapered hole is regarded as the volume variation of the piezoelectric vibrator of the valveless piezoelectric pump, and the micro-tapered hole approximates the conical flow path of the valveless piezoelectric pump. The working principle of the dynamic cone angle is similar to that of a diffuser/nozzle flow tube valveless piezoelectric pump [19].

### 2.3. Analysis of Flow Resistance

In the previous section, we have already explained that the micro-tapered hole is divided into sections I, II, and III. The hole can be seen as a superposition of three tapered flow tubes. According to Huang et al. [20], the flow resistance of the multistage flow tubes can be regarded as the addition of all of the flow resistance. Therefore, the total flow resistance of the flow channel can be superposed by the flow resistance of the three tapered flow tubes.

We defined the instantaneous forward and reverse flow resistances of parts I, II, and III as follows, $\xi_{I}{\left(\chi \right)}_{Sd}$, $\xi_{I}{\left(\chi \right)}_{Sn}$, $\xi_{II}{\left(\chi \right)}_{Sd}$, $\xi_{II}{\left(\chi \right)}_{Sn}$, $\xi_{III}{\left(\chi \right)}_{Sd}$, and $\xi_{III}{\left(\chi \right)}_{Sn}$. *S_d_* is the direction of the forward flow (from III to I) of the liquid, and *S_n_* is the direction of the reverse flow (from I to III).

The total instantaneous forward and reverse flow resistances of the micro-tapered hole are as follows:

(1) $$\xi {\left(\chi \right)}_{Sd}=\xi_{I}{\left(\chi \right)}_{Sd}+\xi_{II}{\left(\chi \right)}_{Sd}+\xi_{III}{\left(\chi \right)}_{Sd}$$

(2) $$\xi {\left(\chi \right)}_{Sn}=\xi_{I}{\left(\chi \right)}_{Sn}+\xi_{II}{\left(\chi \right)}_{Sn}+\xi_{III}{\left(\chi \right)}_{Sn}$$

Under the AC signal, the volume and the taper angle of the micro-tapered hole are periodically changed under the action of the piezoelectric vibrator, and the change in the taper angle will cause the forward and reverse flow resistances to change. We therefore used the average flow resistance to represent the flow resistance of the micro-tapered holes during a cycle.

In a cycle, the average forward and reverse flow resistances of the micro-tapered hole are, as follows:

(3) $$\begin{array}{l} {\bar{\xi (\chi )}}_{Sd}=\frac{\int_{\chi \to \infty }\xi {\left(\chi \right)}_{Sd}d\chi }{\chi }\\ =\frac{\int_{\chi \to \infty }\left(\xi_{I}{\left(\chi \right)}_{Sd}+\xi_{II}{\left(\chi \right)}_{Sd}+\xi_{III}{\left(\chi \right)}_{Sd}\right)d\chi }{\chi } \end{array}$$

(4) $$\begin{array}{l} {\bar{\xi (\chi )}}_{Sn}=\frac{\int_{\chi \to \infty }\xi {\left(\chi \right)}_{Sn}d\chi }{\chi }\\ =\frac{\int_{\chi \to \infty }\left(\xi_{I}{\left(\chi \right)}_{Sn}+\xi_{II}{\left(\chi \right)}_{Sn}+\xi_{III}{\left(\chi \right)}_{Sn}\right)d\chi }{\chi } \end{array}$$

**Figure 4:**
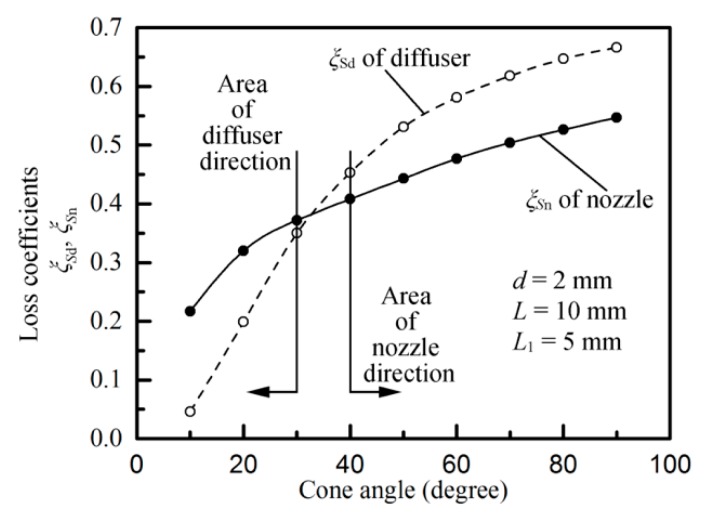
Empirical curves of the cone angle, the diffuser loss coefficient *ξS_d_*, and the nozzle loss coefficient *ξS_n_*.

We discuss only the case in which the angle of the micro-tapered hole is greater than 40°. Figure 4 shows the empirical curves of the cone angle, the diffuser loss coefficient *ξS_d_*, and the nozzle loss coefficient *ξS_n_* [21]. Figure 4 shows that when the angle of the micro-tapered hole is greater than 30°, the flow resistance of the gradually expanding flow is greater than that of the tapered flow. Therefore, the flow through the tapered tube is greater than that of the gradually expanding tube under the same conditions. Here, ${\bar{\xi (\chi )}}_{Sd}$ > ${\bar{\xi (\chi )}}_{Sn}$.

### 2.4. Atomization Rate

In this research, we considered only the atomization rate caused by the volume variation in the micro-tapered hole. According to the results of previous research [15,16], the volume of the micro-tapered hole changes as follows:

(5) $$\begin{array}{cc} \Delta V_{d}= & \underset{\Omega }{∭}[2z(f_{yy}+f_{xx}+f_{y}^{2}f_{xx}\\ & -2f_{x}f_{y}f_{xy}+f_{x}^{2}f_{yy})m^{-1}]dV \end{array}$$

As shown by Cai et al. [15], z[z = *f* (*x*, *y*)] is a neutral surface after deformation, *m* = 1 + *f_x_*^2^ + *f_y_*^2^ + *f_z_*^2^, and *Ω* is the triple integral, which is used to calculate the volume of the micro-aperture hole change.

Therefore, according to the flow equation of the valveless piezoelectric pump, the atomization rate can be calculated using Equation (6), as follows:

(6) $$Q=\Delta V_{d}fn\frac{{\bar{\xi (\chi )}}_{Sd}-{\bar{\xi (\chi )}}_{Sn}}{2+{\bar{\xi (\chi )}}_{Sd}+{\bar{\xi (\chi )}}_{Sn}}$$

where Δ*V_d_* is the volume change in the micro-aperture hole, *f* is the working frequency, and *n* is the number of apertures. From the atomization equation, we find that changes in the micro-aperture hole volume, the operating frequency, and the flow resistance affect the atomization rate.

## 3. Design of Experiments

In this study, the liquid used in the experiment was water, which had a temperature of 20 °C and a kinematic viscosity of 1 × 10^−3^ Pa·s. In this paper, we designed a new experiment to directly measure the atomization rate caused by the volume variation in the micro-tapered hole. During atomization, the atomizer piece collects water via a cotton stick. The contact surface between the atomizer piece and the cotton stick is directly connected with the external environment. When the volume of the piezoelectric vibrator changes during vibration, the atmosphere can be exchanged continuously with the external environment, thereby eliminating the possibility that the atomization is caused by the pressure increase. Therefore, in this experiment, atomization occurred exclusively via the volume variation in the micro-tapered hole.

**Figure 5:**
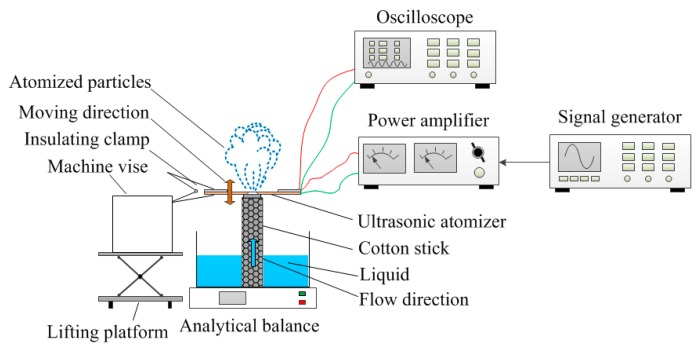
Schematic of the atomization rate measurement.

Figure 5 shows a schematic of the atomization measurement. Insulating clamps were used to mount the atomizer piece, and the insulating clamp was fixed by a machine vise, which was placed on the lifting platform. The height of the atomizer piece was adjusted by the lifting platform, and the atomizer piece only touched the cotton stick (there was no gap or applied force between the sheet and the swab). The orange arrow indicates the direction of movement of the atomizer piece. The cotton stick was placed in a container filled with liquid. Because of the capillary force action, the liquid flowed from the container to the atomizer piece, in the direction of the blue arrow. During operation, the continuous volume variation in the micro-tapered hole produced a pumping effect to move the liquid from the cotton stick to the external environment, thereby producing the atomization phenomenon. In this experiment, we adjusted the signal generator employed to output the AC voltage and used the power amplifier to amplify the AC voltage. The amplified AC voltage was applied to the atomizer, and the AC voltage was monitored by an oscilloscope. During the measurement of the atomization rate, the atomizer was placed on a high-precision analytical balance, and a stopwatch was used to time the power supply voltage to the atomizer. We stopped supplying the voltage to the atomizer after 1 min, and the atomization rate was determined by measuring the reduction of liquid in the liquid chamber per minute, using the high-precision analytical balance.

**Figure 6:**
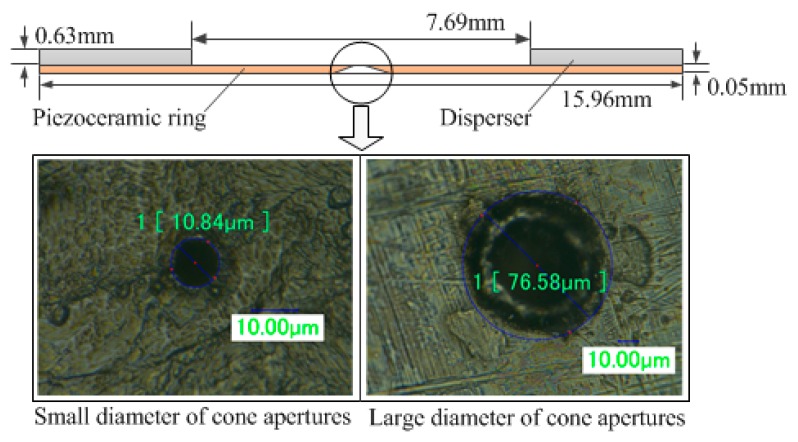
The structure and parameters of the atomizer piece.

Figure 6 shows the parameters of the atomizer piece used in this study. The specific parameters of the atomizer piece are as follows. The outer diameter, inner diameter, and thickness of the piezoelectric ceramic ring were 15.96 mm, 7.69 mm, and 0.63 mm, respectively, and the diameter and thickness of the disperser were 15.96 mm and 0.05 mm, respectively. The small diameter and large diameter of the cone apertures were 10.84 µm and 76.58 µm, respectively (the diameter of the cone apertures was measured via a microscope). The optimal operating frequency of the atomizer piece was 121.1 kHz (optimal operating frequency was measured by a laser vibrometer).

The experiment was carried out at a room temperature (20 °C) and at an atmospheric pressure of 101,325 Pa. The type of signal generator used in this experiment was a Tektronix AFG 3022B (Manufacturer: Tektronix, Beaverton, OR, USA). The power amplifier was an HFVA-105A (Manufacturer: Nanjing Buddha science and Technology Industry Co., Ltd, Nanjing, China), and the oscilloscope was a Tektronix DPO2014 (Manufacturer: Tektronix, USA).

**Figure 7:**
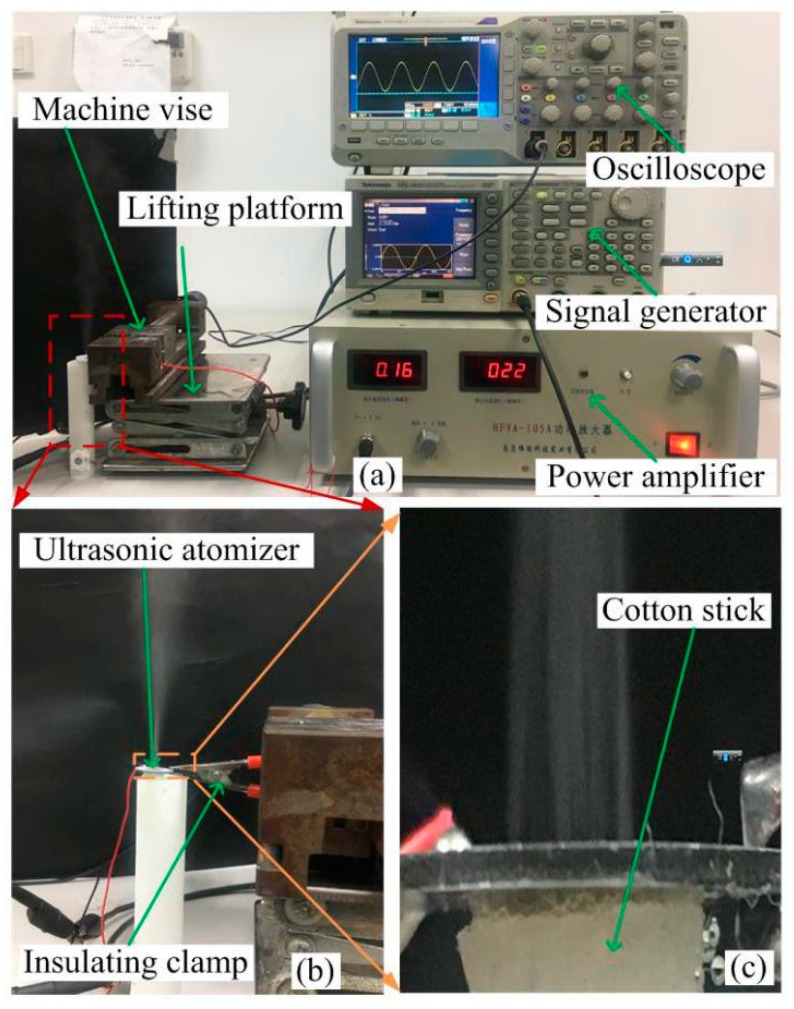
Photograph of the atomization rate measurement apparatus. (**a**) Photograph of the atomization rate measurement apparatus; (**b**) A partial enlarged view of figure (**a**); (**c**) A partial enlarged view of figure (**b**).

Figure 7a shows a photograph of the atomization rate measurement apparatus, and the content of the photograph is the same as that of Figure 7a. Figure 7b shows a partial enlarged view of Figure 7a, and Figure 7c shows a partially enlarged view in Figure 7b. Figure 7b shows the clamping between the insulating clamp and the atomizer piece, and Figure 7c shows the contact between the cotton stick and the atomizer piece.

## 4. Results and Discussion

**Figure 8:**
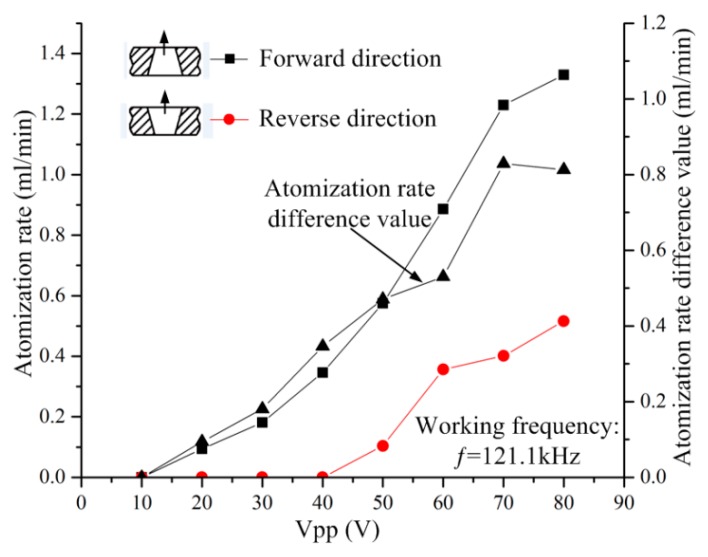
Variation in the atomization rate and the atomization rate and differences with applied voltage when the micro-tapered aperture atomizer is operating in the forward and reverse directions.

The atomization rates were measured at both the flared (forward-direction) and tapered (reverse-direction) sides under different applied voltages, at 121.1 kHz, and the differences were calculated and plotted, as shown in Figure 8. The atomization rate was measured according to the atomization rate measurement schematic, shown in Figure 5. The following observations were made during the measurement procedure. No visible atomization was observed in either the forward or reverse directions when the voltage was less than 20 V. For the micro-tapered aperture operating in the forward direction, visible atomization was generated when the applied voltage exceeded 20 V, and the atomization rate increased when the applied voltage increased. For the micro-tapered aperture operating in the reverse direction, only water drops were formed, even when the voltage was as high as 50 V. However, when the voltage reached 50 V, atomization began, and the atomization rate also increased when the applied voltage increased. The atomization rate measured in the forward direction was much higher than that measured in the reverse direction. This atomization rate difference increased as the applied voltage increased. In this study, the atomization rate was caused by the volume variation in the micro-tapered hole.

In this experiment, the atomization rate measured in the forward direction was much higher than that measured in the reverse direction, indicating that the micro-tapered aperture played an important role in the atomization process. The difference in the atomization rate was mainly caused by the difference between the forward and reverse direction flow resistances of the micro-tapered hole. We measured the atomization rates in the forward and reverse directions and found that they increase as the applied voltage increases. This effect is observed because the volume variation rate of the micro-tapered hole increases with the increase in voltage. With the increase in the volume variation rate of the micro-tapered hole, the pumping effect [17] and the difference between the forward and reverse direction flow resistances of the micro-tapered hole become more significant. According to Formula (6), the micro-tapered hole caused the increased atomization rate.

To further measure the atomization rate generated by each micro-tapered hole and the influence of the volume of the micro-tapered hole on the atomization rate, we processed the single micro-tapered hole atomizer piece with three different parameters, as shown in Table 1. The remaining parameters of the three types of atomizer plates were consistent with those of the previous porous atomizer plates.

**Figure 9:**
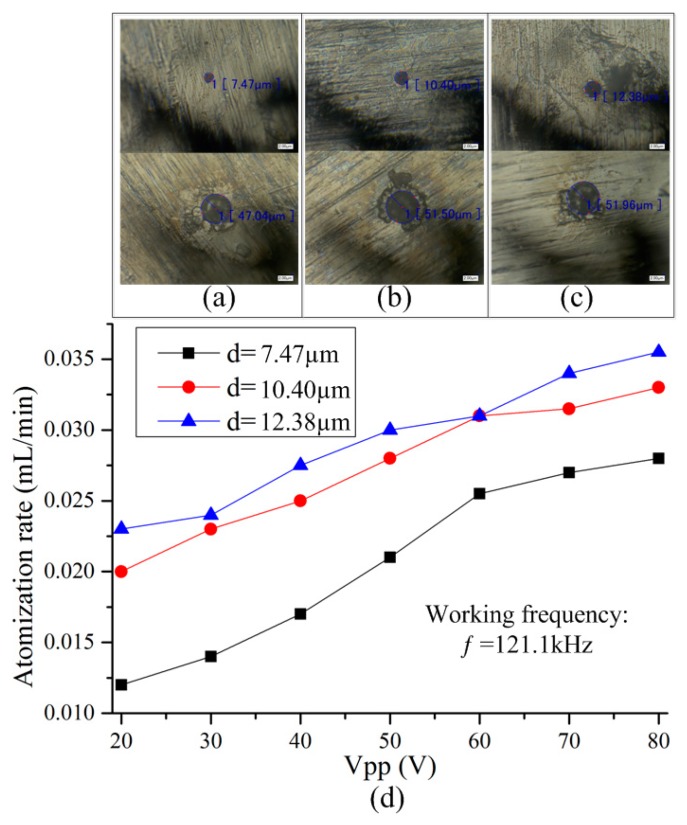
The relationship between the atomization rate and voltage for three micro-tapered aperture atomizers with different diameters. (**a**) Photographs of the micro-tapered holes with number 1; (**b**) Photographs of the micro-tapered holes with number 2; (**c**) Photographs of the micro-tapered holes with number 3; (**d**) The relationship between the atomization rate and voltage for three micro-tapered aperture atomizers with different diameters.

Figure 9a–c are photographs of the micro-tapered holes on the three types of atomizer plates, taken with a microscope. There is only one micro-tapered hole in each atomizing plate. When the remaining parameters of the atomizer plates were the same, the order of the volume of the three types of micro-tapered holes was as follows: Vc > Vb > Va. Figure 9d shows the relationship between the atomization rate and voltage for the three micro-tapered aperture atomizers with different diameters. No visible atomization was observed in the three different micro-tapered aperture atomizers when the voltage was less than 20 V. For the micro-tapered aperture atomizer, visible atomization was generated when the applied voltage exceeded 20 V, and the atomization rate increased when the applied voltage increased. The atomization rate measured was caused by the volume change in the micro-tapered hole.

We measured the atomization rate of three single-diameter atomizers of different sizes, and found that they increase as the applied voltage increases. This effect is observed because the volume variation rate of the micro-tapered hole increases with the increase in voltage. With the increase in the volume variation rate of the micro-tapered hole, the pumping effect [17] and the difference between the forward and reverse direction flow resistances of the micro-tapered hole become more significant. According to Formula (6), the micro-tapered hole caused the increased atomization rate. Under the same voltage, the atomization rate increased with increasing volume of the micro-tapered hole. This phenomenon occurred because the deformation of the dispenser was the same under the same operating voltage, and because the volume variation in the micro-tapered hole increased with increasing micro-tapered hole volume, causing the pumping effect of the micro-tapered hole to become more significant. Therefore, the atomization rate induced by the pumping effect also increased.

## 5. Conclusions

This paper summarizes the previous research results of our research team, explains the pump effect of the micro-tapered hole, and derives the atomization rate equation. We also designed an atomization measurement experiment that eliminated the atomization caused by increased pressure, and demonstrated that the volume change in a micro-tapered hole could produce atomization. The experimental results show that the forward atomization rate is much higher than the reverse atomization rate. This is because the flow resistance plays an important role in atomization. We measured the atomization rates in the forward and reverse directions and found that they increase as the applied voltage increases. This is because the volume variation rate of the micro-tapered hole increases with the increase in voltage, and the pumping effect becomes more significant with the increase in the volume variation rate of the micro-tapered hole. Measuring the atomization rate of three single-diameter atomizers of different sizes, led to the finding that the volume of the micro-tapered hole also plays an important role in atomization. This study is expected to aid in the popularization of the micro-aperture atomizer in inhalation therapy.

## Figures and Tables

**Table 1:** Diameters of the three types of micro-tapered holes.

Number	1	2	3
Small diameter [μm]	7.47	10.40	12.38
Large diameter [μm]	47.04	51.50	51.96
